# The Endocrine Disrupting Compounds Bisphenol‐A and α‐Zeranol Mimic the Estrogen Transcriptional Program to Promote Proliferation and Stemness in Breast Cancer Cells

**DOI:** 10.1002/mc.70127

**Published:** 2026-05-03

**Authors:** Cassandra Winz, Eric Li, Caroline Xie, Kyo Chang Lee, Kevin Boguszewski, Shlok Rohatagi, Rita Hahn, Ray Rancourt, Philip Furmanski, Nanjoo Suh

**Affiliations:** ^1^ Department of Chemical Biology, Ernest Mario School of Pharmacy, Rutgers The State University of New Jersey Piscataway New Jersey USA; ^2^ Joint Graduate Program in Toxicology at Rutgers The State University of New Jersey Piscataway New Jersey USA; ^3^ Ernest Mario School of Pharmacy at Rutgers The State University of New Jersey Piscataway New Jersey USA; ^4^ Rutgers Cancer Institute of New Jersey New Brunswick New Jersey USA

**Keywords:** breast cancer, cancer stem cells, endocrine disrupting compounds, estrogen

## Abstract

Excessive activation of the estrogen receptor (ER) drives proliferation, progression, and the formation of breast cancer stem cells (CSCs) in ER‐positive breast cancer. Estrogenic endocrine disrupting compounds (EDCs) found in plastics, water, and food are also able to bind to the ER. Thus, we hypothesized that estrogenic EDCs mimic estrogen (E2) in the pathogenesis of breast cancer by promoting their survival and proliferation. Three estrogenic EDCs routinely found in human biosamples were selected for analysis: bisphenol‐A (BPA), diethyl‐hexyl phthalate (DEHP), and alpha‐zeranol (αZAL). We assessed proliferation, transcriptional reprogramming, and CSC formation in breast cancer cell lines. E2, BPA, and αZAL significantly increased cell proliferation in ER‐positive, but not ER‐negative cell lines. This was reversed after administration of the ER‐antagonist, ICI 182,780. BPA and αZAL upregulated estrogen target genes (PGR, TFF1) and increased levels of cell‐cycle protein. RNA sequencing analysis revealed that BPA and αZAL altered expression of genes related to cell division, DNA repair, and estrogen signaling, with a substantial transcriptional overlap between EDCs and estrogen treatments. Additionally, BPA and αZAL increased the proportion of CSCs, defined as the CD24^low^/CD44^high^ expressing subpopulation. Overall, these data indicate that BPA and αZAL act as functional estrogen mimics in breast cancer cells, activating canonical estrogen signaling pathways and promoting stem‐like characteristics. Notably, this study provides the first transcriptomic and stem‐associated characterization of αZAL in ER‐positive breast cancer cells, revealing a robust estrogenic mode of action. This work provides mechanistic insight into how environmental EDCs may influence ER‐positive breast cancer biology.

**Abbreviations**
aZAL: alpha‐zeranol
BPA: bisphenol‐A
CSC: cancer stem cell
DEHP: diethyl‐hexyl phthalate
DMSO: dimethyl sulfoxide
E2: beta‐estradiol
EDC: endocrine disrupting compounds
ER: estrogen receptor
MEHP: monoethyl‐hexyl phthalate
PGR: progesterone receptor
TFF: trefoil factor

## 1 Introduction

In the United States, one in eight women will be diagnosed with breast cancer in their lifetime [1]. The incidence of breast cancer is increasing globally, and most diagnosed cases have no attributable inherited cause [2]. Excessive stimulation of estrogen signaling pathways is a hallmark of estrogen receptor‐positive (ER‐positive) breast cancer, a breast cancer subtype that makes up 80% of all diagnosed cases [3, 4]. ER‐positive breast cancer cells rely on estrogen stimulation to survive, progress, and metastasize. Importantly, estrogen signaling promotes the formation of cancer stem cells (CSCs), a quiescent, therapy‐resistant subpopulation capable of driving tumor metastasis and recurrence [5]. CSCs from ER‐positive breast cancers retain expression of the estrogen receptor and thus remain responsive to estrogenic stimuli [6, 7]. Higher CSC populations in ER‐positive breast cancers are associated with worse outcomes and increased mortality in patients [8].

Exposure to endocrine disrupting compounds (EDCs) may play a large role in breast cancer incidence and progression [9]. The increasing use of EDCs in industrial manufacturing, as well as their presence in contaminated foodstuffs, makes exposure to the human population widespread [10]. Estrogenic EDCs share chemical similarity with the endogenous sex steroid hormone estrogen (e.g., 17β‐estradiol or E2). Given that estrogen is the primary driver of ER‐positive breast cancer, estrogenic EDCs may similarly promote cancer progression in this subtype. While many studies have assessed an association between EDC exposures and breast cancer incidence [11, 12], few have assessed a link between exposure to EDCs and poorer ER‐positive breast cancer prognosis and mortality. The role of these endocrine disruptors in promoting already existing breast cancers may be just as important as their role in initiation. Therefore, we aimed to assess whether exposure to selected estrogenic EDCs induces breast cancer cell proliferation and stemness in an ER‐dependent manner.

As EDCs represent a diverse class of compounds with varying sources, structures, and potentially different biological activities, we selected three estrogenic EDCs from different structural classes: bisphenol‐A (BPA), a widely studied plasticizer found in consumer products; alpha‐zeranol (aZAL), an understudied mycotoxin contaminant of food crops; and diethyl‐hexyl phthalate (DEHP), a moderately characterized plasticizer [13, 14]. These compounds are commonly detected in human biosamples and have demonstrated estrogenic activity in vitro [15, 16, 17, 18, 19, 20]. While BPA's effects in breast cancer cells are well‐established [21], the breast cancer‐specific activities of aZAL and DEHP remain poorly characterized, representing important knowledge gaps for risk assessment. In this study, breast cancer cells were treated with these EDCs, and estrogen signaling pathways, proliferation, and cancer stem cell (CSC) populations were assessed. RNA sequencing technologies were employed to assess global changes to the overall transcriptome induced by EDCs. This work provides mechanistic insight into how environmentally relevant EDCs may influence ER‐positive breast cancer progression through estrogen receptor‐dependent pathways.

## 2 Materials and Methods

### 2.1 Cell Culture and Reagents

**Figure 1:**
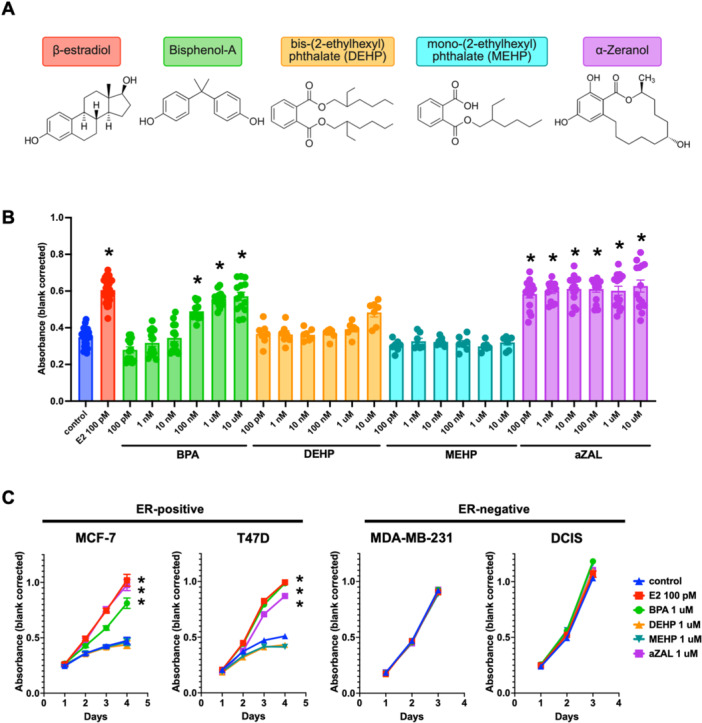
EDCs promote cell proliferation in ER‐positive breast cancer cell lines. (A) Chemical structures of main test compounds. (B) MCF‐7 cells were seeded as previously described and treated with EDC at concentrations ranging from 100 pM to 10 μM. Three independent replicates were performed. Data represent mean ± SEM (*n* = 3) (C) MCF‐7 and T47D were seeded into 96 well plates at a cell count of 2,000 cells/well while DCIS and MDA‐MB‐231 were seeded at a count of 1000 cells/well. MTT assays were performed at 24, 48, 72, and 96 h post exposure. Significance was determined via one‐way ANOVA (*n* = 4). A * denotes that *p* < 0.05.

17β‐estradiol (E2) was obtained from Sigma‐Aldrich (St. Louis, MO; #E2758) and dissolved in dimethyl sulfoxide (DMSO) at a stock solution concentration of 10 mM and stored at −20°C for cell culture use. BPA (Sigma‐Aldrich #239658), DEHP (Sigma‐Aldrich #36735), MEHP (Sigma Aldrich #796832), and aZAL (Fujifilm #262‐01461) were dissolved in DMSO at a concentration of 100 mM and stored at −20°C for cell culture use (Figure 1A). The human ER‐positive breast cancer cell lines MCF‐7 and T47D, as well as the ER‐negative cell line MDA‐MB‐231 were acquired from American Type Culture Collection (ATCC, Manassas, VA) and maintained in DMEM/F12 medium supplemented with 10% fetal bovine serum. The human ER‐negative breast cancer MCF10DCIS.com (DCIS) cell line was provided by Dr. Fred Miller at the Barbara Ann Karmanos Cancer Institute (Detroit, MI) and maintained in DMEM/F12 medium supplemented with 5% horse serum. All cell lines were kept at 37°C and 5% CO_2_ and were authenticated using Short Tandem Repeat profiling (Cat# 135‐XV) at American Type Culture Collection (ATCC) on November 21, 2025. Before treatment, cells were seeded in complete growth medium and incubated overnight. The following day, cells were switched to phenol red‐free RPMI medium supplemented with 5% charcoal‐stripped FBS and treated as indicated.

### 2.2 Cell Proliferation Assays

MCF‐7 and T47D cells were seeded into 96‐well plates at a cell count of 2000 cells/well, while DCIS and MDA‐MB‐231 cells were seeded at a cell count of 1000 cells/well. For pharmacologic inhibition studies, MCF‐7 cells were seeded into 24‐well plates at a cell count of 4000 cells/well. MTT assays were carried out as previously described [22].

### 2.3 Quantitative Polymerase Chain Reaction

RNA quality was assessed prior to downstream analyses by measuring absorbance ratios using a Tecan microplate reader. Samples with 260/280 ratios between 1.8 and 2.0 and sufficient RNA concentration were accepted for use. The procedure was described previously [23]. The primers (*CCND1* #Hs00277039, ENSG00000110092; *CD44* #Hs01075861, ENSG00000026508; *C‐MYC* #Hs00153408, ENSG00000136997; *GAPDH* #Hs02758991, ENSG00000111640; *NFKB* #Hs00765730, ENSG00000109320; *PGR* #Hs01556702, ENSG00000082175; *SERPINA1* #Hs00165475, ENSG00000197249; *TFF1* #Hs00907239, ENSG00000160182) were obtained from Applied Biosystems (Pleasonton, CA). The ddCt values were calculated from Ct values compared to GAPDH, the reference gene. Results are represented as fold changes compared to the control group.

### 2.4 Western Blot Analysis

The detailed procedures have been described previously [24]. The primary antibody detecting c‐myc (1:1000, #5605) and β‐actin, (1:2000 #A1978) were from Cell Signaling Technology (Danvers, MA) and Sigma‐Aldrich (St. Louis, Missouri), respectively. Secondary antibodies were obtained from Cell Signaling Technology.

### 2.5 Bulk RNA Sequencing

RNA sequencing was performed using the MCF‐7 cell line, based on its ER‐positivity and responsiveness to estrogen and the estrogen disrupters. Monolayer MCF‐7 cells were treated with DMSO (0.01%), estrogen (100 pM), BPA (1 μM), or aZAL (1 μM) were harvested at 12 and 72 h post‐treatment, and RNA was isolated. Prior to library preparation, RNA sample quality was verified by Novogene Co. Ltd. (Sacramento, CA) using a combination of RIN scoring, spectrophotometric purity assessment (A260/280 and A260/230 ratios), and library fragment size and yield analysis, with all samples meeting accepted thresholds for sequencing. Doses were selected based on prior cell viability data indicating that the 1 μM concentration produced robust biological responses without cytotoxicity. Bulk sequencing was performed by Novogene using an Illumina NovaSeq. 6000 platform to generate paired‐end reads. Raw sequencing data quality was assessed, and reads were aligned to the human reference genome GRCh38/hg38 followed by gene‐level quantification to generate count matrices. All statistical analyses were performed in R (version 4.5.1) using the DESeq. 2 package (version 1.48.2). Gene count matrices were filtered to remove lowly expressed genes, retaining only genes with at least 10 counts in at least 3 samples. Size factors were estimated to account for differences in library sizes across samples, and dispersion parameters were estimated using the DESeq. 2 empirical Bayes shrinkage approach. Differential expression testing was performed using the Wald test implemented in DESeq. 2. For each comparison, genes were considered differentially expressed if they had an adjusted p‐value (FDR) < 0.05 after Benjamini‐Hochberg correction for multiple testing. Log2 fold changes were calculated relative to control conditions. Principal component analysis (PCA) was performed on variance‐stabilized transformed (VST) count data to assess overall sample relationships and identify potential batch effects. Hierarchical clustering was performed using Euclidean distance and complete linkage to evaluate sample similarity. Heatmaps were generated using the pheatmap package to visualize expression patterns of differentially expressed genes across all samples and treatment conditions. Gene symbols and annotations were obtained using biomaRt packages. Functional enrichment analysis was performed using Gene Ontology and KEGG pathway analysis to identify biological pathways and processes associated with differentially expressed genes. The RNA‐Seq datasets described in this study have been deposited in the NCBI Gene Expression Omnibus with accession number GSE311462.

### 2.6 Tumorsphere Forming Assay

Monolayer MCF‐7 cells were dissociated using accutase (Corning, NY) and seeded into an ultra‐low attachment plate (Corning, NY) in tumorsphere media. Tumorsphere media comprised of MEBM, 20 ng/mL fibroblast growth factor, 20 ng/mL epidermal growth factor, heparin, insulin from bovine pancreas, and 1X B‐27 supplement as previously described [25]. Monolayer DCIS cells were seeded in tumorsphere media containing MammoCult media (Cell Signaling, NY), MammoCult supplement, 0.2% heparin, and hydrocortisone 2.5 µg/mL. Cells were treated at the time of seeding for 5 days, upon which resulting tumorspheres were imaged using a TE200 microscope (Nikon Instrument, Melville, NY) and RNA was harvested for subsequent qPCR analysis.

### 2.7 Flow Cytometry

Tumorsphere suspension cultures were spun down and incubated with accutase for 6 min. Cells were washed with BD Pharmingen Stain Buffer (FBS) and stained with antibodies against CD44 APC or CD24 PE from BD Biosciences (San Jose, CA) for 1 h. The scatter properties and fluorescent intensities of stained cells were measured on a Galios flow cytometer (Beckman Coulter) using a logarithmic scale for data collection at a cell count of 1 × 10^5^ per 200 µL of PBS. Ten thousand total events were collected and the populations of CD24^low^/CD44^high^ and CD24^high^/CD44^low^ were assessed and compared amongst the treatment groups using Kaluza software. The mean fluorescent intensities (MFI) for FL2 (PE: Excitation 488 nm/Emission 575/20 nm) and FL6 (APC: Excitation 633 nm/Emission 660/20 nm) were plotted against the number of collected events.

### 2.8 Statistical Analysis

All statistical significance was assessed by a one‐way ANOVA statistical test, followed by a Tukey test. A value of *p* < 0.05 was considered statistically significant. Results have been visualized using Prism 10 (La Jolla, CA) or R (version 4.5.1).

## 3 Results

### 3.1 BPA and aZAL Promote Cell Proliferation of ER‐Positive but not ER‐Negative Breast Cancer Cells

To assess the cell proliferative effects of the selected EDCs at increasing concentrations, a dose‐response assay was carried out in the ER‐positive MCF‐7 cell line. BPA significantly increased cell proliferation at concentrations between 100 nM and 10 μM, while αZAL increased proliferation at a broader concentration range of 100 pM to 10 μM. No differences in cell proliferation were seen after exposure to DEHP. Since DEHP is rapidly metabolized to MEHP in humans [26], we also treated cells with MEHP, which similarly had no effect on cell proliferation in vitro (Figure 1B).

Next, a panel of breast cancer cell lines was treated with fixed EDC concentrations. In ER‐positive cell lines (MCF‐7, T47D), E2 (100 pM), BPA (1 μM), and αZAL (1 μM) significantly increased cell proliferation after 96‐h, while DEHP (1 μM) and MEHP (1 μM) had no effect. ER‐negative cell lines (DCIS, MDA‐MB‐231) were not affected by E2 or EDC treatment (Figure 1C). These results indicates that BPA and αZAL promote breast cancer cell proliferation in an ER‐dependent manner.

### 3.2 ER Antagonist ICI‐182,780 Blocks EDC‐Induced Cell Proliferation

**Figure 2:**
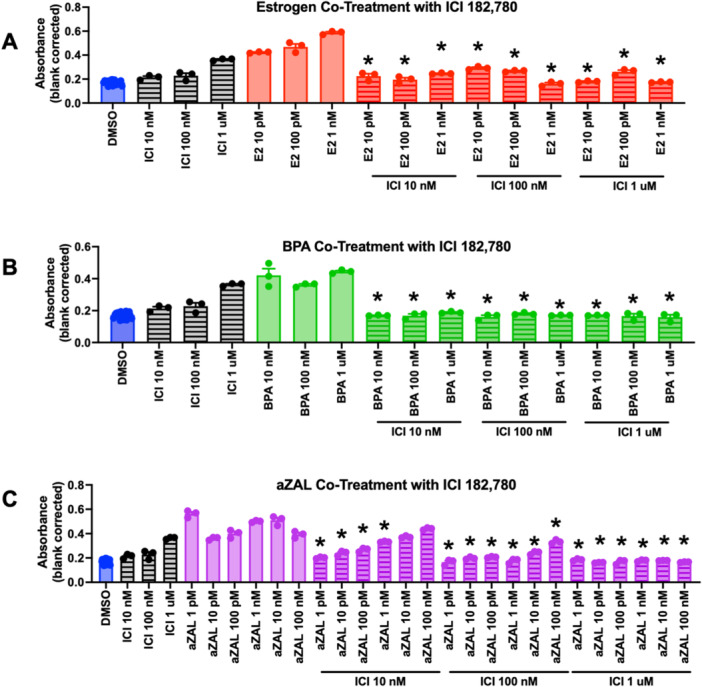
Proliferative effects of BPA and aZAL are blunted by co‐treatment with ICI‐182,780. MCF‐7 cells were seeded as previously described and treated with (A) E2, (B) BPA, or (C) aZAL. ICI‐182,780 was co‐administered at the time of treatment at doses ranging from 10 nM to 1 μM. An MTT assay was conducted 96 h after treatment. Significance was determined via one‐way ANOVA (*n* = 3). A * denotes that *p* < 0.05 for the comparison of ICI‐182,780 + E2/EDC treatment compared to E2/EDC alone.

To further assess the ER‐dependency of EDC‐induced breast cancer proliferation, MCF‐7 cells were co‐treated with ICI‐182,780, a pharmacologic inhibitor of the ERα. Cells were treated with increasing concentrations of estrogen (10 pM to 1 nM), BPA (10 nM to 1 μM), or αZAL (1 pM to 100 nM). ICI‐182,780 concentrations ranged from 10 nM to 1 μM. As DEHP and MEHP did not alter cell proliferation, only BPA and αZAL were investigated in all further analyses. We found that after 96 h, estrogen‐induced cell growth was ameliorated by co‐treatment with ICI‐182,780 (Figure 2A). Similarly, BPA‐induced cell growth was reversed with ICI‐182,780, indicating that the ER is likely the primary driver of cell growth (Figure 2B). Notably, αZAL‐induced proliferation was more resistant to ICI‐182,780 inhibition. At a low dose of ICI‐182,780, higher concentrations of αZAL still induced cell proliferation (Figure 2C).

### 3.3 Transcriptomic Profiling of ER‐Positive Breast Cancer Cells Exposed to BPA and aZAL

**Figure 3:**
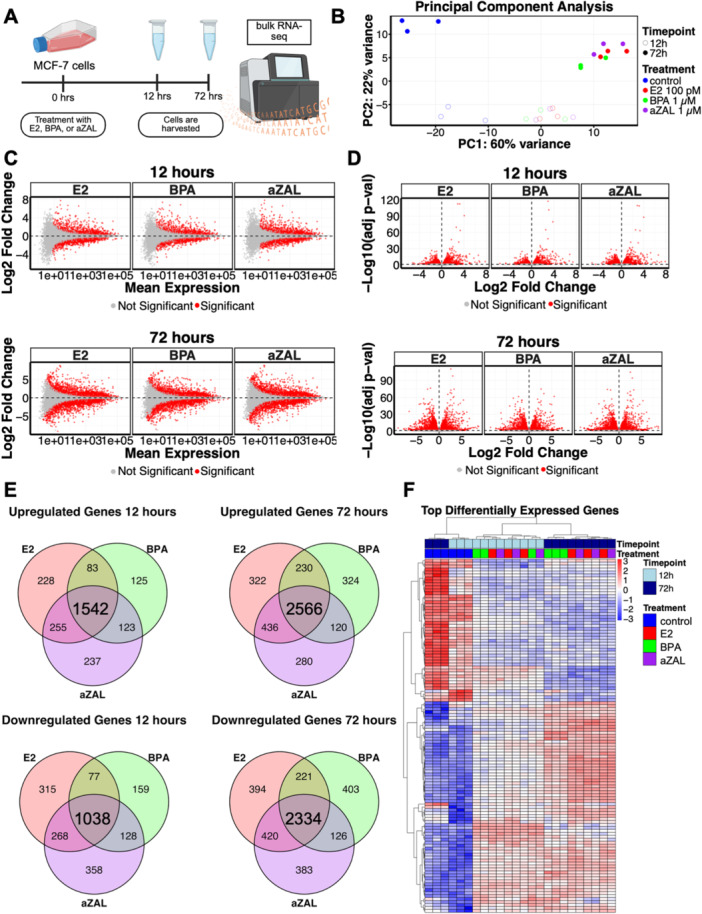
Transcriptome profiling reveals shared programs among E2 and EDCs. (A) MCF‐7 cells were seeded into flasks at a cell count of 250 K cells and treated with DMSO 0.01%, E2 100 pM, BPA 1 μM, or aZAL 1 μM. Total RNA was extracted 12 or 72 h after treatment and bulk RNA sequencing was performed by Novogene. Three biological replicates were included in analysis. (B) Principal component analysis (PCA) of normalized gene expression demonstrates clear separation by timepoint and treatment. (C) MA plots show log2 fold changes of all genes versus mean expression for each treatment at 12 h (top) and 72 h (bottom). Red dots signify significantly differentially expressed genes, where FDR < 0.05. Differential expression analysis was performed using DESeq. 2 with Benjamini‐Hochberg FDR correction. Genes with |log2 fold change | ≥ 1 and FDR < 0.05 were considered significantly differentially expressed. (D) Volcano plots display statistical significance (‐log10 adjusted *p*‐value) versus fold change for each treatment and timepoint. (E) Venn diagrams demonstrate the overlap of significantly upregulated (top) and downregulated (bottom) genes between E2, BPA, and aZAL at both timepoints. (F) The top differentially expressed genes shared amongst the three treatment groups were clustered in a heat map based on Euclidean distance. Rows represent genes while columns represent samples grouped by treatment and timepoint.

To determine if EDCs promoted the same transcriptional effects as E2, bulk RNA‐sequencing was performed on MCF‐7 cells treated with DMSO 0.01%, E2 (100 pM), BPA (1 μM), or αZAL (1 μM) for 12 or 72 h (*n* = 3 per group) (Figure 3A). The 1 μM concentration for BPA and αZAL was selected based on the initial dose‐response cell proliferation assay to ensure sufficient transcriptional activation for the detection of differentially expressed genes and to enable direct comparison between compounds. Principal component analysis revealed that samples clustered based on treatment and timepoint, with PC1 (60% variance) separating treatment conditions and PC2 (22% variance) separating timepoint. Additionally, BPA and αZAL samples clustered together with E2, indicating their similar transcriptional profiles (Figure 3B).

MA and volcano plots revealed that significant transcriptional reprogramming events occurred in the E2, BPA, and αZAL treatment groups (Figure 3C and 3D). Using |log2FC | ≥ 1 and FDR < 0.05 as significance thresholds, more genes were differentially expressed at 72 h than at 12 h. At 12 h, E2, BPA, and αZAL significantly altered the expression of 2123, 1887, and 2185 genes, respectively. By 72 h, the number of significantly altered genes rose to 6780, 6257, and 6555.

There was extensive overlap between transcriptomic profiles of E2, BPA, and αZAL‐treated breast cancer cells. At 12 h, the three treatments upregulated the same 1542 of 2132 total upregulated genes and downregulated the same 1038 of 1870 total downregulated genes. By 72 h, the overlap among the three treatments increased to 2566 of 3492 upregulated genes and 2334 of 3513 downregulated genes (Figure 3E).

Unsupervised hierarchical clustering of the top differentially expressed genes revealed clear separation of samples by both treatment and timepoint. Control samples clustered away from treatment samples independent of timepoint, whereas within the estrogen, BPA, and αZAL samples, a clear timepoint effect was seen (Figure 3F). Within treatments, BPA clustered separately from E2 and αZAL at both timepoints, suggesting partially distinct transcriptional responses despite overall similarity. Over time, genes became more strongly differentially expressed, with some reversing expression entirely. This points to a clear temporal relationship in the alteration of the breast cancer transcriptome by both estrogen and endocrine disruptors.

### 3.4 BPA and αZAL Induce Estrogen‐Responsive Genes and Proliferative Pathways

**Figure 4:**
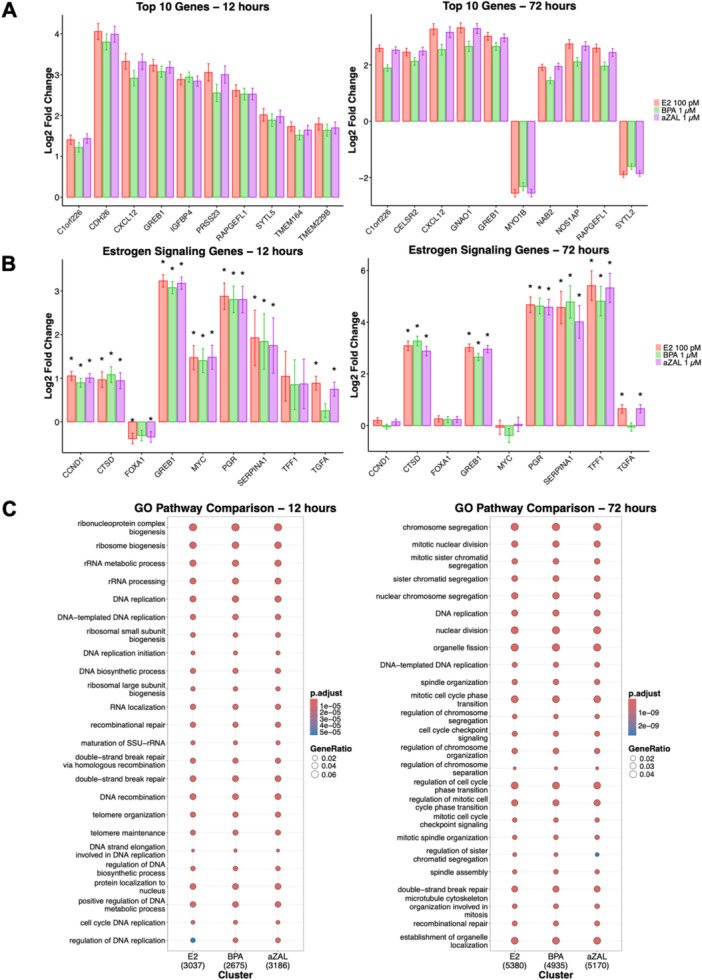
BPA and aZAL upregulate programs related to estrogen signaling and cancer cell turnover. Bar graphs denote the expression profiles of the top 10 most significantly differentially expressed genes (A) and classical estrogen‐responsive genes (B) at 12 h (left) and 72 h (right). Error bars represent standard error of the mean amongst three biological replicates. (C) Gene ontology biological process pathway enrichment analysis is demonstrated via bubble plots of significantly enriched pathways (FDR < 0.05) for each treatment at 12 h (left) and 72 h (right). Bubble size represents the proportion of genes differentially expressed in a given pathway and color represents the significance. GO pathway enrichment performed using clusterProfiler with Benjamini‐Hochberg adjustment for multiple comparisons.

To identify affected pathways shared between estrogen, BPA, and αZAL, we examined the expression patterns of the top 10 most altered genes at both the 12 and 72‐h timepoints. Several of these genes have been linked to breast cancer progression, such as *CDH26*, *IGFBP4*, *CELSR2*, and *NOS1AP* (Figure 4A). Notably, Growth Regulation by Estrogen Binding Protein 1 (*GREB1*) was among the top 10 most altered genes at both the 12 and 72 h timepoints, pointing to a shared canonical estrogen receptor‐mediated signaling pathway.

Next, we assessed the expression of several estrogen responsive genes. At the 12 h timepoint, many of these genes were already upregulated, but by 72 h, the estrogenic responses were more pronounced and consistent across all treatments. PGR, a quintessential estrogen target gene, was significantly upregulated by E2, BPA, and αZAL, with all three treatments showing similar fold changes (Figure 4B). Similarly, GREB1, TFF1, FOXA1, and MYC demonstrated the same pattern of differential gene expression by all three compounds. These changes were even more evident at the 72 h timepoint. Notably, SERPINA1 and CTSD also showed treatment‐induced changes, further confirming the activation of estrogen signaling pathways by both EDCs.

The biological processes affected by estrogen, BPA, and αZAL treatment were further assessed by performing gene ontology (GO) on differentially expressed genes. At both the 12 and 72 h timepoint, the top enriched pathways were remarkably similar among the treatment groups. Dominant pathways included chromosome segregation, mitotic nuclear division, DNA replication, and regulation of cell cycle phase transition, indicating strong activation of proliferative programs (Figure 4C). These biological processes are important for estrogen‐mediated cancer cell proliferation and survival.

Collectively, these data demonstrate that BPA and αZAL function as potent estrogen mimics in ER‐positive breast cancer cells, activating both individual estrogen target genes and broader transcriptional programs associated with estrogen‐driven proliferation.

### 3.5 BPA and aZAL Upregulate Estrogen Target Genes and Proliferation Markers

**Figure 5:**
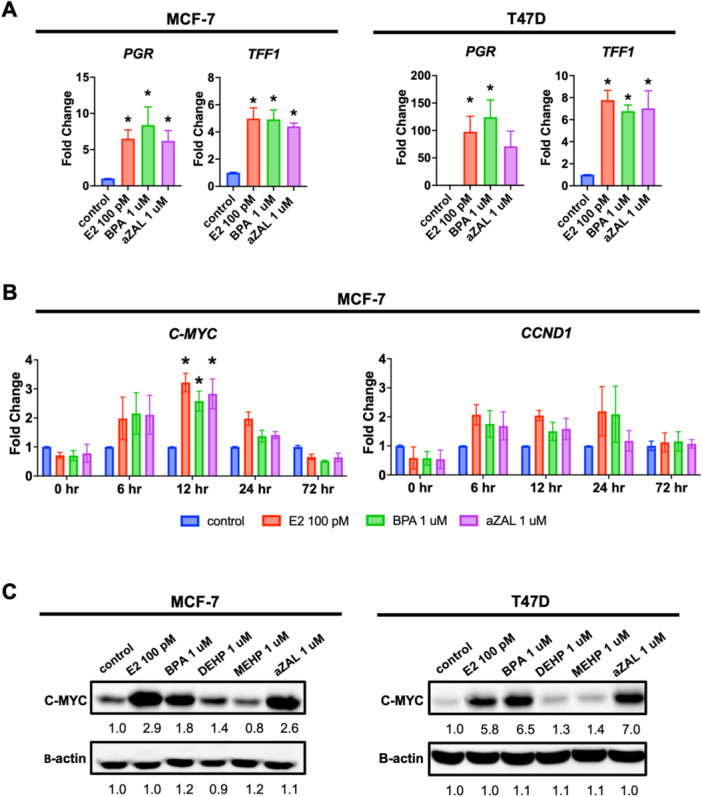
EDCs increase expression of ER target markers and cell cycle genes in ER‐positive breast cancer cell lines. (A) MCF‐7 and T47D cells were seeded into flasks at a cell count of 250 K cells. qPCR analysis was carried out to determine the gene expression levels of PGR and TFF1 in both cell lines. (B) MCF‐7 cells were treated with E2 (100 pM), BPA (1 μM), or aZAL (1 μM) and harvested at timepoints up to 72 h. Expression levels of CMYC and CCND1 were assessed. All significance was determined via One‐way ANOVA. A * denotes a *p* < 0.05 compared to timepoint controls. (C) Western blot analysis was carried out for C‐MYC. Blots were quantified via FIJI/ImageJ. Representative images shown.

qPCR was utilized to validate the effects seen with RNA‐seq and to assess the expression of estrogen‐signaling molecules in ER‐positive breast cancer cells. The expression of *PGR* and *TFF1* was significantly induced following treatment with BPA or αZAL (1 μM each) at 48 h in ER‐positive cell lines (MCF‐7, T47D) (Figure 5A). The expression of the cell proliferation markers *C‐MYC* and *CCND1* were determined following exposure to 1 μM of BPA or αZAL in the MCF‐7 cell line at 0, 6, 12, 24, and 72 h post‐treatment. Expression of *C‐MYC* peaked 12 h post‐treatment, resulting in significant upregulation in the E2, BPA, and αZAL treatment groups, while expression of *CCND1* showed a trending increase at the 12‐h time point (Figure 5B). This was further confirmed at the protein level using western blotting, where 100 pM of estrogen or 1 μM of BPA or αZAL for 24 h increased the level of C‐MYC in MCF‐7 and T47D cell cultures (Figure 5C).

### 3.6 BPA and αZAL Enhance Cancer Stem Cell Characteristics

**Figure 6:**
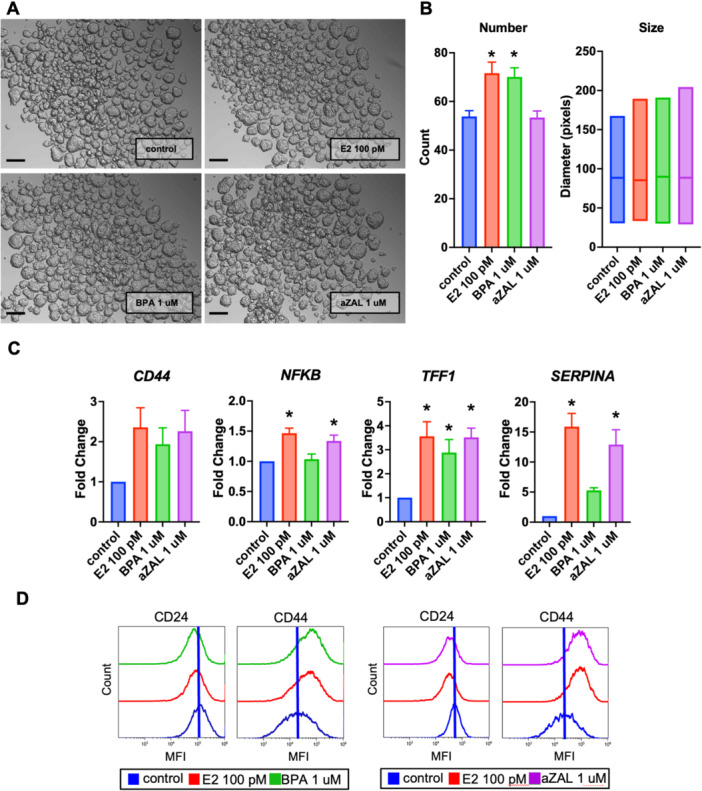
EDCs increase cancer stem cell markers in ER‐positive breast cancer tumorspheres. MCF‐7 cells were plated at a count of 5000 cells/well in ultra‐low attachment 24‐well plates and grown for 5 days in the presence of E2 100 pM, BPA 1 μM, or aZAL 1 μM, and subsequently imaged (*n* = 3). (A) Representative images shown. (B) Resulting spheres were counted and their diameters were measured using FIJI/ImageJ. Bars represent 100 μm. (C) qPCR was carried out on resulting mammospheres to assess the expression of CD44, NFKB, SERPINA1, and TFF1. Bars signify mean +/− SEM. Significance determined via one‐way ANOVA. A * denotes a *p* < 0.05. (*n* = 3). (D) Cells were stained with combinations of antibodies against CD44 and CD24, and flow cytometry was performed. Histograms show the mean fluorescence intensity of CD44 (PE) and CD24 (APC). Representative histograms shown (*n* = 3).

We next examined breast cancer stem cells (CSCs) in BPA and αZAL‐treated cell culture. To assess cancer stemness, the in vitro tumorsphere formation model was utilized. This culturing technique uses nutrient‐poor, low‐attachment conditions. The differentiated, rapidly proliferating cells that make up the bulk of the cancer cells in both breast tumors and cell culture will not survive in this culture [27]. Surviving cancer stem cells form suspended colonies, called “tumorspheres,” that can be enumerated and characterized. MCF‐7 cells were treated with E2 (100 pM), BPA (1 μM), or αZAL (1 μM) in tumorsphere conditions for 5 days, and were subsequently imaged (Figure 6A). Resulting tumorspheres were counted and their diameters were measured using ImageJ (Figure 6B). BPA significantly increased the number of resulting tumorspheres in the MCF‐7 cell line. However, this change was not seen in the ER‐negative breast cancer cell line DCIS, though αZAL did significantly increase the number of resulting tumorspheres in this cell line (Supporting Information Figure S1).

To further assess the effects of EDCs on cancer stemness in ER‐positive breast cancer, qPCR analysis was carried out on treated tumorspheres. CD44 was increased in the estrogen, BPA, and αZAL treatment groups, and NFKB was significantly upregulated in both the estrogen and αZAL treatment groups. Additionally, TFF1, a key estrogen signaling marker, was significantly upregulated in the E2, BPA, and αZAL treatment groups, while SERPINA was upregulated in the E2 and αZAL treatment groups (Figure 6C).

Lastly, flow cytometry was carried out in MCF‐7 tumorspheres to assess the subpopulation of cells with a cancer stem cell phenotype. CSCs are characterized by a CD24^low^/CD44^high^ molecular signature. BPA and αZAL shifted the subpopulation of cancer cells such that they had more CD44 expression and less CD24 expression. E2 treatment produced similar effects (Figure 6D, Supporting Information Figure S2).

### 3.7 IPA Mapping Reveals Similarities Between Estrogen, BPA, and αZAL by Pathway

**Figure 7:**
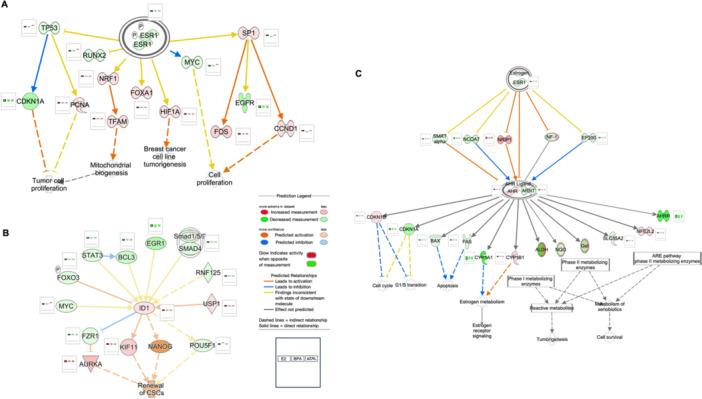
Gene expression mapping onto IPA canonical pathways reveals similar effects of estrogen, BPA, and aZAL. MCF‐7 cells were treated with estrogen, BPA, or aZAL for 72 h, and differentially expressed genes were mapped onto canonical pathways using Ingenuity Pathway Analysis (IPA). Pathways displayed include (A) the estrogen receptor pathway, (B) cancer stem cell signaling, and (C) the aryl hydrocarbon receptor pathway. Red shading indicates upregulation, while green shading indicates downregulation, determined via *p*‐value (−1.3 > logp > 1.3). Orange arrows represent a predicted activation relationship, while blue arrows represent a predicted inhibition relationship. Yellow arrows indicate an unpredicted result.

IPA pathway analysis mapped gene expression data to the canonical estrogen receptor pathway for estrogen, BPA, and αZAL treated MCF‐7 cells at 72 h (Figure 7A, Supporting Information Figures S3, S4, and S5). Notably, BPA and αZAL had the same effects as estrogen on most molecules in the pathway, with few exceptions. These differentially expressed pathways are associated with downstream processes such as tumor cell proliferation, mitochondrial biogenesis, and cell proliferation. Gene expression data from this study was also mapped to a cancer stem cell molecular pathway in the IPA database (Figure 7B). Similarly, all three treatments had remarkably similar effects to this pathway. Lastly, the gene expression data was mapped to the aryl hydrocarbon receptor (AhR) downstream pathway (Figure 7C). The AhR has frequent cross‐talk with the estrogen receptor, and has been cited as a potential target of BPA [28]. Differential gene expression for each of the molecules in the pathway were identical amongst the estrogen, BPA, and αZAL treatment groups, indicating a remarkably similar mechanism of action.

## 4 Discussion

In the present study, BPA and αZAL behave as estrogen mimics in ER‐positive breast cancer cell lines. This effect appears to be mediated by canonical estrogen receptor activation, as it does not occur in ER‐negative breast cancer cell lines or when ER+ breast cancers are co‐treated with ER antagonists. Further, BPA and αZAL shared many of the same transcriptional programs as estrogen. Lastly, assessments of the stemness of treated breast cancer tumorspheres indicate that EDCs increase the cancer stem‐like subpopulation in culture. This suggests that BPA and αZAL may enhance stem‐like characteristics in ER‐positive breast cancer cells, potentially influencing breast cancer disease outcomes.

The present study utilized both monolayer and 3D in vitro cell culture of several key breast cancer cell lines. The major limitation of this model is that monoculturing does not recapitulate the complete complex tumor microenvironment. We used a single ER+ breast cancer cell line for whole transcriptome analysis. To ameliorate this limitation, alternative ER+ cell lines, namely, T47D, were used for other endpoints.

A critical consideration in interpreting these findings is the concentration‐response relationship observed for each compound. In the initial dose response assay, BPA increased ER‐positive breast cancer cell proliferation at doses from 10 nM to 10 μM, while αZAL promoted ER‐positive cell proliferation at doses as low as 1 pM. These differential response patterns may reflect distinct binding affinities for ERα, as αZAL (IC50 = 2.18 × 10⁻⁸ M) demonstrates higher ER binding affinity than BPA (IC50 = 1.03 × 10⁻⁶ M) and approaches that of estradiol (IC50 = 1.04 × 10⁻⁸ M) [29, 30].

RNA sequencing analysis revealed extensive transcriptional overlap between EDCs and E2 treated MCF‐7 cells. The activation of classical estrogen targets (PGR, TFF1, GREB1) and proliferation‐associated pathways (cell cycle regulation, DNA replication, chromosome segregation) by BPA and αZAL confirms canonical ER pathway engagement. In both PCA and unsupervised hierarchical clustering, BPA clustered apart from estrogen and αZAL. This could also point to alternative actions between BPA and αZAL, perhaps explained by their differing binding affinities for ERα. Alternatively, other mechanisms have been postulated, including noncanonical estrogen receptor activation, such as GPR30 [31].

A single dose (1 μM) was used for both BPA and αZAL in RNA sequencing experiments to capture the full transcriptional program activated by each EDC and facilitate direct comparison between the compounds. This dose, identified in the initial dose response screens, ensured adequate statistical power to detect differentially expressed genes. Urinalysis and serum analysis of human study participants have found concentrations of αZAL ranging from 10 to 100 nM [19, 32] and of BPA ranging from 1 to 10 nM [33]. While our proliferation data demonstrate that BPA and αZAL act as estrogen mimics to increase breast cancer proliferation at exposure‐relevant concentrations, our transcriptomic and stem cell data utilize doses exceeding human exposure levels. However, the primary objective at this point was to identify potentially deleterious modes of action and to characterize pathway‐level mechanisms of estrogenic EDCs, rather than to quantify extrapolated human exposures.

A limitation of these transcriptomic analyses is the use of three biological replicates per group, which is standard for RNA‐seq studies in controlled cell line systems but may limit statistical power to detect subtle transcriptional changes of smaller magnitude. Post‐hoc power analysis using RnaSeqSampleSize, based on empirical parameters from our dataset (dispersion = 0.039, average read counts = 155.9), demonstrated that *n* = 3 per group provides 92% power to detect twofold changes at FDR = 0.05, confirming adequate power for robust transcriptional responses. Power was substantially reduced (25%) for more modest 1.5‐fold changes, suggesting that subtle transcriptional effects may not be fully captured. These findings are consistent with the study's primary objective of identifying pathway‐level estrogenic programs rather than exhaustively cataloging all transcriptional changes.

To assess cancer stem cell (CSC) activity, both functional stemness (tumorsphere formation) and phenotypic marker expression (CD44+ /CD24−) were evaluated, as these measures are not always concordant [34]. BPA significantly increased the number, but not the size, of MCF‐7 mammospheres, while αZAL did not consistently alter either the size or number of spheres in culture. However, both compounds consistently enriched the CD44+ /CD24‐ cell population in MCF‐7 cells, as detected by flow cytometry. The observed discordance between functional stem cell activity and phenotypic marker enrichment may suggest that BPA and αZAL exposure alter plasticity without immediately enhancing self‐renewal. Consistent with this interpretation, IPA analysis of monolayer cultures revealed that estrogen, BPA, and αZAL similarly affected CSC signaling pathways. Thus, the observed CD44+ /CD24− marker enrichment and upregulated CSC transcriptional pathways may represent early reprogramming events that could favor later enhanced cancer stem cell function under permissive conditions. Such increases are clinically relevant, as cells with increased plasticity are associated with therapeutic resistance [35, 36]. Future studies should examine whether similar effects occur at environmentally relevant concentrations and determine whether prolonged exposure affects functional endpoints, such as self‐renewal capacity.

BPA has been researched extensively as a potential xenoestrogen in several in vitro ER+ breast cancer models. The results of the present study concur with previously established literature demonstrating that, at similar doses, BPA behaves as a xenoestrogen in MCF‐7 cell culture [37, 38, 39]. While BPA has been well‐established as a breast cancer mitogen in culture, its potential cancer stem cell‐promoting effects are understudied [40]. One study found that treatment with BPA induced CSC characteristics, like tumorsphere size and stem cell gene expression [41]. Our findings further confirm this phenotype by using flow cytometry to identify and enumerate the cancer stem cell population in ER‐positive tumorspheres after BPA treatment.

In contrast to the scope of BPA research, insight into αZAL and other zearalenone metabolites regarding breast cancer is scarce. Existing studies have identified αZAL as a potential breast cancer mitogen, primarily through proliferation studies [42, 43]. To our knowledge, the present study is the first to characterize the estrogen‐responsive transcriptional landscape and stem‐associated phenotypic effects of αZAL in ER‐positive breast cancer cells. By directly comparing BPA and αZAL across multiple endpoints, this work identifies shared and distinct estrogenic modes of action and highlights the potential for agriculturally derived EDCs to influence breast cancer biology. These findings provide mechanistic insight into how EDCs might influence ER+ breast cancer biology, but future research priorities include studies at environmentally relevant concentrations to determine threshold effects, the use of patient‐derived organoid models to improve physiological relevance, and functional validation of stemness effects. Our work contributes to understanding EDC biology and the research presented provides a foundation for more comprehensive risk assessment strategies that consider both chemical structure and biological activity patterns across different EDC classes.

## 5 Conclusion

In the absence of relevant clinical data, characterizing the molecular mechanisms through which EDCs may influence ER+ breast cancer is important for informing risk assessment and supporting future epidemiological studies. Our results demonstrate that BPA and αZAL activate canonical estrogen receptor signaling pathways in ER+ breast cancer cells, promoting proliferation and enhancing stem‐like characteristics through ER‐dependent mechanisms. These findings reveal that structurally distinct EDCs can converge on similar transcriptional programs, activating estrogen target genes and proliferation‐associated pathways. Notably, αZAL demonstrated potent estrogenic activity despite limited previous characterization in breast cancer models. However, the concentrations required for transcriptomic and stemness effects substantially exceed typical human exposure levels, limiting the translational relevance of these mechanistic insights. Future studies examining these effects at environmentally relevant concentrations are essential to determine whether such mechanisms operate under realistic exposure scenarios. This work provides a foundation for more comprehensive risk assessment strategies that consider both chemical diversity and dose‐response relationships across EDC classes.

## Author Contributions

Cassandra Winz conceived and designed the experiments, performed experiments and data analysis, visualization and validation, and wrote the initial draft of the article. Eric Li and Caroline Xie contributed significantly to the experiments and data analysis. Kyo Chang Lee, Kevin Boguszewski, Shlok Rohatagi, Rita Hahn, and Ray Rancourt contributed to the experiments and data analyzes. Philip Furmanski conceived and designed the experiments and revised the final article. Nanjoo Suh conceived and designed the experiments, visualization and data arrangement, and wrote the final article. Cassandra Winz, Philip Furmanski, and Nanjoo Suh revised the final article. All authors read and approved the final article.

## Ethics Statement

The authors confirm that there are no animal or human studies conducted in this study.

## Conflicts of Interest

The authors declare no conflicts of interest.

## Supporting information

Supporting File 1

Supporting File 2

Supporting File 3

Supporting File 4

Supporting File 5

## Data Availability

The data that support the findings of this study are available from the corresponding author upon reasonable request. The RNA‐Seq datasets described in this study have been deposited in the NCBI Gene Expression Omnibus with accession number GSE311462. The datasets generated during and/or analyzed during the current study are included in the manuscript and can be available from the corresponding author upon reasonable request.

## References

[1] N. Howlader , A. N , M. Krapcho , et al., SEER Cancer Statistics Review, 1975–2018. 2021, NCI.

[2] A. N. Giaquinto , H. Sung , K. D. Miller , et al., “Breast Cancer Statistics, 2022,” CA: A Cancer Journal for Clinicians 72, no. 6 (2022): 524–541. 36190501 10.3322/caac.21754

[3] W. Yue , J. D. Yager , J. P. Wang , E. R. Jupe , and R. J. Santen , “Estrogen Receptor‐Dependent and Independent Mechanisms of Breast Cancer Carcinogenesis,” Steroids 78, no. 2 (2013): 161–170. 23178278 10.1016/j.steroids.2012.11.001

[4] A. N. Giaquinto , H. Sung , K. D. Miller , et al., “Breast Cancer Statistics, 2022,” CA: A Cancer Journal for Clinicians 72, no. 6 (2022): 524–541. 36190501 10.3322/caac.21754

[5] Y.‐S. Lu , G. X. Yao , X. L. Wang , et al., “A Comprehensive Analysis of Metabolomics and Transcriptomics Reveals New Biomarkers and Mechanistic Insights on DEHP Exposures in MCF‐7 Cells,” Chemosphere 255 (2020): 126865. 32402870 10.1016/j.chemosphere.2020.126865

[6] B. Chen , P. Ye , Y. Chen , et al., “Involvement of the Estrogen and Progesterone Axis in Cancer Stemness: Elucidating Molecular Mechanisms and Clinical Significance,” Frontiers in Oncology 10 (2020): 1657. 33014829 10.3389/fonc.2020.01657 PMC7498570

[7] Y. Sun , Y. Wang , C. Fan , et al., “Estrogen Promotes Stemness and Invasiveness of ER‐Positive Breast Cancer Cells Through Gli1 Activation,” Molecular Cancer 13, no. 1 (2014): 137. 24889938 10.1186/1476-4598-13-137 PMC4057898

[8] H. E. Lee , J. H. Kim , Y. J. Kim , et al., “An Increase in Cancer Stem Cell Population After Primary Systemic Therapy is a Poor Prognostic Factor in Breast Cancer,” British Journal of Cancer 104, no. 11 (2011): 1730–1738. 21559013 10.1038/bjc.2011.159 PMC3111169

[9] P. D. Darbre , “Chapter Thirteen ‐ Endocrine Disrupting Chemicals and Breast Cancer Cells.” in Advances in Pharmacology, eds. L. N. Vandenberg and J. L. Turgeon . (Academic Press, 2021), 485–520. 10.1016/bs.apha.2021.04.006 34452695

[10] E. Diamanti‐Kandarakis , J. P. Bourguignon , L. C. Giudice , et al., “Endocrine‐Disrupting Chemicals: An Endocrine Society Scientific Statement,” Endocrine Reviews 30, no. 4 (2009): 293–342. 19502515 10.1210/er.2009-0002 PMC2726844

[11] P. R. S. Rocha , V. D. Oliveira , C. I. Vasques , P. E. D. dos Reis , and A. A. Amato , “Exposure to Endocrine Disruptors and Risk of Breast Cancer: A Systematic Review,” Critical Reviews in Oncology/Hematology 161 (2021): 103330. 33862246 10.1016/j.critrevonc.2021.103330

[12] G. M. Calaf , R. Ponce‐Cusi , F. Aguayo , J. P. Munoz , and T. C. Bleak , “Endocrine Disruptors From the Environment Affecting Breast Cancer,” Oncology Letters 20, no. 1 (2020): 19–32. 10.3892/ol.2020.11566 PMC7286136 32565930

[13] Z. Wang and Y. Wang , “Mechanism Exploration of Di(2‐Ethylhexyl) Phthalate (DEHP)‐Induced Breast Cancer via Network Toxicology and Molecular Docking Analysis,” Scientific Reports 15, no. 1 (2025): 27257. 40715557 10.1038/s41598-025-13201-1 PMC12297483

[14] L. Yang , X. Liu , Z. Peng , et al., “Exposure to Di‐2‐Ethylhexyl Phthalate (DEHP) Increases the Risk of Cancer,” BMC Public Health 24, no. 1 (2024): 430. 38341560 10.1186/s12889-024-17801-w PMC10859012

[15] A. M. Calafat , Z. Kuklenyik , J. A. Reidy , S. P. Caudill , J. Ekong , and L. L. Needham , “Urinary Concentrations of Bisphenol A and 4‐Nonylphenol in a Human Reference Population,” Environmental Health Perspectives 113, no. 4 (2005): 391–395. 15811827 10.1289/ehp.7534 PMC1278476

[16] P. Perez , R. Pulgar , F. Olea‐Serrano , et al., “The Estrogenicity of Bisphenol A‐Related Diphenylalkanes With Various Substituents at the Central Carbon and the Hydroxy Groups,” Environmental Health Perspectives 106, no. 3 (1998): 167–174. 9449681 10.1289/ehp.98106167 PMC1533034

[17] H. A. A. M. Dirven , P. H. H. van den Broek , and F. J. Jongeneelen , “Determination of Four Metabolites of the Plasticizer di(2‐ethylhexyl)phthalate in Human Urine Samples,” International Archives of Occupational and Environmental Health 64, no. 8 (1993): 555–560. 8314613 10.1007/BF00517700

[18] C. A. Harris , P. Henttu , M. G. Parker , and J. P. Sumpter , “The Estrogenic Activity of Phthalate Esters In Vitro,” Environmental Health Perspectives 105, no. 8 (1997): 802–811. 9347895 10.1289/ehp.97105802 PMC1470189

[19] I. Matraszek‐Zuchowska , B. Wozniak , and J. Zmudzki , “Determination of Zeranol, Taleranol, Zearalanone, α‐Zearalenol, β‐Zearalenol and Zearalenone in Urine by LC‐MS/MS,” Food Additives and Contaminants: Part A 30, no. 6 (2013): 987–994. 10.1080/19440049.2013.787656 23705928

[20] D. G. Lindsay , “Zeranol‐A ‘Nature‐Identical’ Oestrogen?,” Food and Chemical Toxicology 23, no. 8 (1985): 767–774. 2931335 10.1016/0278-6915(85)90273-x

[21] Z. Wang , H. Liu , and S. Liu , “Low‐Dose Bisphenol A Exposure: A Seemingly Instigating Carcinogenic Effect on Breast Cancer,” Advanced Science 4, no. 2 (2017): 1600248. 28251049 10.1002/advs.201600248 PMC5323866

[22] J. Y. So , A. K. Smolarek , D. M. Salerno , et al., “Targeting CD44‐STAT3 Signaling by Gemini Vitamin D Analog Leads to Inhibition of Invasion in Basal‐Like Breast Cancer,” PLoS One 8, no. 1 (2013): e54020. 23326564 10.1371/journal.pone.0054020 PMC3543376

[23] T. Xie , H. Zahid , A. R. Ali , et al., “Inhibitors of Keap1‐Nrf2 Protein‐Protein Interaction Reduce Estrogen Responsive Gene Expression and Oxidative Stress in Estrogen Receptor‐Positive Breast Cancer,” Toxicology and Applied Pharmacology 460 (2023): 116375. 36634873 10.1016/j.taap.2023.116375 PMC9879264

[24] M. J. Bak , S. Das Gupta , J. Wahler , et al., “Inhibitory Effects of γ‐ and δ‐Tocopherols on Estrogen‐Stimulated Breast Cancer In Vitro and In Vivo,” Cancer Prevention Research 10, no. 3 (2017): 188–197. 28096236 10.1158/1940-6207.CAPR-16-0223 PMC5337152

[25] M. J. Bak , P. Furmanski , N. L. Shan , et al., “Tocopherols Inhibit Estrogen‐Induced Cancer Stemness and OCT4 Signaling in Breast Cancer,” Carcinogenesis 39, no. 8 (2018): 1045–1055. 29846560 10.1093/carcin/bgy071 PMC6067126

[26] H. M. Bolt , J. Angerer , and H. M. Koch , “Di(2‐ethylhexyl)phthalate (DEHP) Metabolites in Human Urine and Serum After a Single Oral Dose of Deuterium‐Labelled DHEP,” Archives of Toxicology 78, no. 3 (2004): 123–130. 14576974 10.1007/s00204-003-0522-3

[27] C. H. Lee , C. C. Yu , B. Y. Wang , and W. W. Chang , “Tumorsphere as An Effective In Vitro Platform for Screening Anti‐Cancer Stem Cell Drugs,” Oncotarget 7, no. 2 (2016): 1215–1226. 26527320 10.18632/oncotarget.6261 PMC4811455

[28] O. Banerjee , S. Singh , S. K. Prasad , et al., “Exploring Aryl Hydrocarbon Receptor (AhR) as a Target for Bisphenol‐A (BPA)‐Induced Pancreatic Islet Toxicity and Impaired Glucose Homeostasis: Protective Efficacy of Ethanol Extract of Centella Asiatica,” Toxicology 500 (2023): 153693. 38042274 10.1016/j.tox.2023.153693

[29] A. Matsushima , X. Liu , H. Okada , M. Shimohigashi , and Y. Shimohigashi , “Bisphenol AF Is a Full Agonist for the Estrogen Receptor ERα but a Highly Specific Antagonist for ERβ,” Environmental Health Perspectives 118, no. 9 (2010): 1267–1272. 20427257 10.1289/ehp.0901819 PMC2944088

[30] H. Takemura , J. Y. Shim , K. Sayama , A. Tsubura , B. T. Zhu , and K. Shimoi , “Characterization of the Estrogenic Activities of Zearalenone and Zeranol In Vivo and In Vitro,” The Journal of Steroid Biochemistry and Molecular Biology 103, no. 2 (2007): 170–177. 17097287 10.1016/j.jsbmb.2006.08.008

[31] S. Dong , S. Terasaka , and R. Kiyama , “Bisphenol A Induces a Rapid Activation of Erk1/2 Through GPR30 in Human Breast Cancer Cells,” Environmental Pollution 159, no. 1 (2011): 212–218. 20875696 10.1016/j.envpol.2010.09.004

[32] D. Sun , C. Li , S. Zhou , et al., “Determination of Trace Zearalenone and Its Metabolites in Human Serum by a High‐Throughput UPLC‐MS/MS Analysis,” Applied Sciences 9, no. 4 (2019): 741.

[33] L. N. Vandenberg , R. Hauser , M. Marcus , N. Olea , and W. V. Welshons , “Human Exposure to Bisphenol A (BPA),” Reproductive Toxicology 24, no. 2 (2007): 139–177. 17825522 10.1016/j.reprotox.2007.07.010

[34] P. C. Bailey , R. M. Lee , M. I. Vitolo , et al., “Single‐Cell Tracking of Breast Cancer Cells Enables Prediction of Sphere Formation From Early Cell Divisions,” iScience 8 (2018): 29–39. 30268511 10.1016/j.isci.2018.08.015 PMC6170521

[35] X. Yuan , K. Chen , F. Zheng , et al., “Low‐Dose BPA and Its Substitute Bps Promote Ovarian Cancer Cell Stemness via a Non‐Canonical PINK1/p53 Mitophagic Signaling,” Journal of Hazardous Materials 452 (2023): 131288. 36989771 10.1016/j.jhazmat.2023.131288

[36] J. P. Muñoz , “The Impact of Endocrine‐Disrupting Chemicals on Stem Cells: Mechanisms and Implications for Human Health,” Journal of Environmental Sciences 147 (2025): 294–309. 10.1016/j.jes.2023.11.015 39003048

[37] R. Mesnage , A. Phedonos , M. Arno , S. Balu , J. C. Corton , and M. N. Antoniou , “Editor's Highlight: Transcriptome Profiling Reveals Bisphenol A Alternatives Activate Estrogen Receptor Alpha in Human Breast Cancer Cells,” Toxicological Sciences 158, no. 2 (2017): 431–443. 28591870 10.1093/toxsci/kfx101 PMC5837682

[38] Z. Awada , R. Nasr , R. Akika , et al., “Dna Methylome‐Wide Alterations Associated With Estrogen Receptor‐Dependent Effects of Bisphenols in Breast Cancer,” Clinical Epigenetics 11, no. 1 (2019): 138. 31601247 10.1186/s13148-019-0725-y PMC6785895

[39] A. Mlynarcikova , L. Macho , and M. Fickova , “Bisphenol A Alone or in Combination With Estradiol Modulates Cell Cycle‐ and Apoptosis‐Related Proteins and Genes in MCF7 Cells,” Endocrine Regulations 47, no. 4 (2013): 189–199. 24156707 10.4149/endo_2013_04_189

[40] C. Winz and N. Suh , “Understanding the Mechanistic Link Between Bisphenol A and Cancer Stem Cells: A Cancer Prevention Perspective,” Journal of Cancer Prevention 26, no. 1 (2021): 18–24. 33842402 10.15430/JCP.2021.26.1.18 PMC8020171

[41] M. A. Lillo , C. Nichols , T. N. Seagroves , G. A. Miranda‐Carboni , and S. A. Krum , “Bisphenol A Induces Sox2 in ER(+) Breast Cancer Stem‐Like Cells,” Hormones and Cancer 8, no. 2 (2017): 90–99. 28244015 10.1007/s12672-017-0286-5 PMC5458606

[42] P. Xu , W. Ye , S. Zhong , et al., “Zeranol May Increase the Risk of Leptin‐Induced Neoplasia in Human Breast,” Oncology Letters 2, no. 1 (2011): 101–108. 22870137 10.3892/ol.2010.214 PMC3412469

[43] S. Zhong , S. Liu , S. Chen , H. Lin , W. Wang , and X. Qin , “Zeranol Stimulates Proliferation and Aromatase Activation in Human Breast Preadipocytes,” Molecular Medicine Reports 14, no. 1 (2016): 1014–1018. 27220457 10.3892/mmr.2016.5293

