# Supplementary figures and images for: The Endocrine Disrupting Compounds Bisphenol‐A and α‐Zeranol Mimic the Estrogen Transcriptional Program to Promote Proliferation and Stemness in Breast Cancer Cells
Source: Mol Carcinog. 2026 May 3;65(8):907–20. doi: 10.1002/mc.70127 (PMC13372423; doi:10.1002/mc.70127)

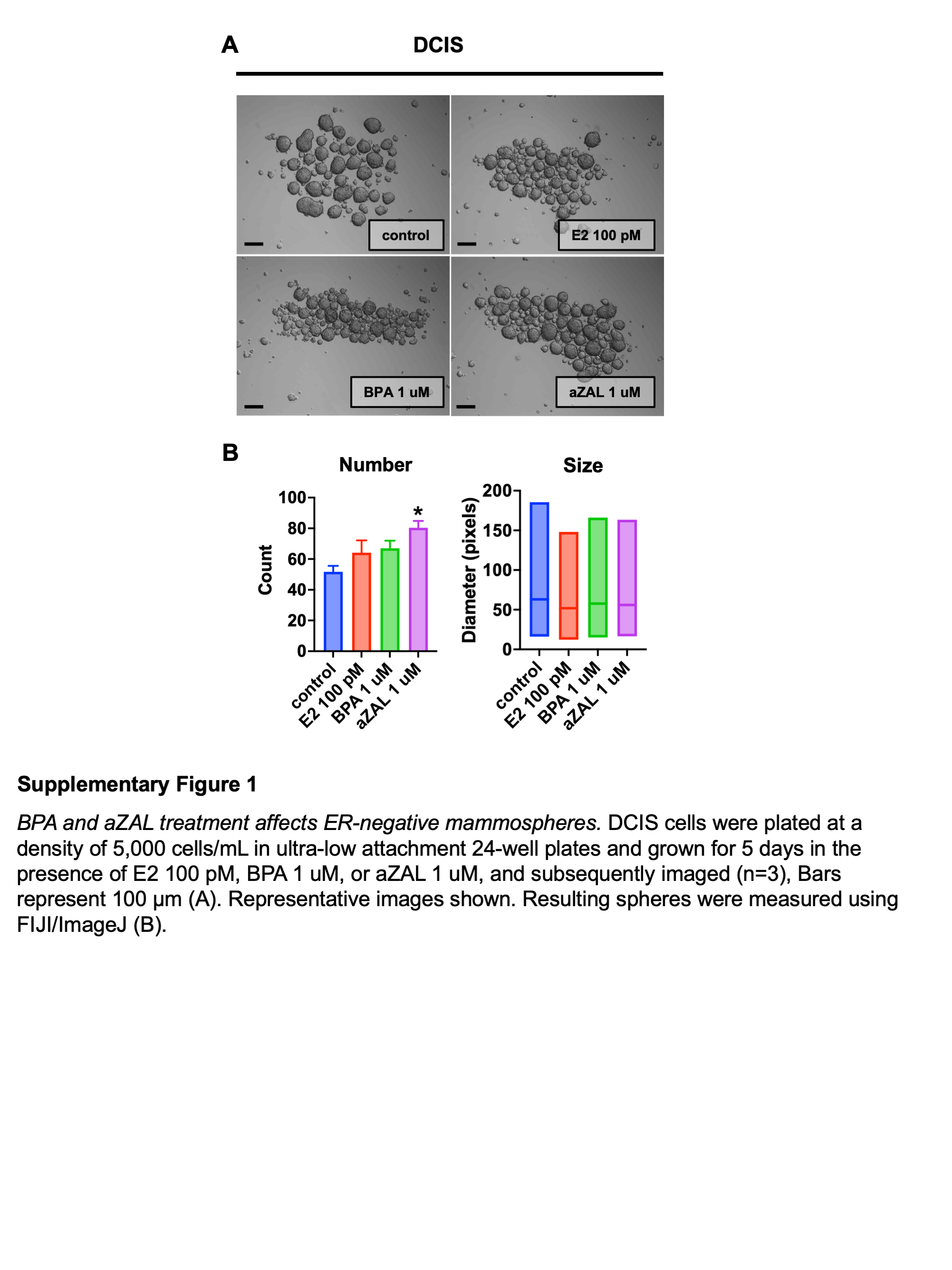

Supplement: Supplementary file 1 — Supporting File 1 [file MC-65-907-s001.tiff]

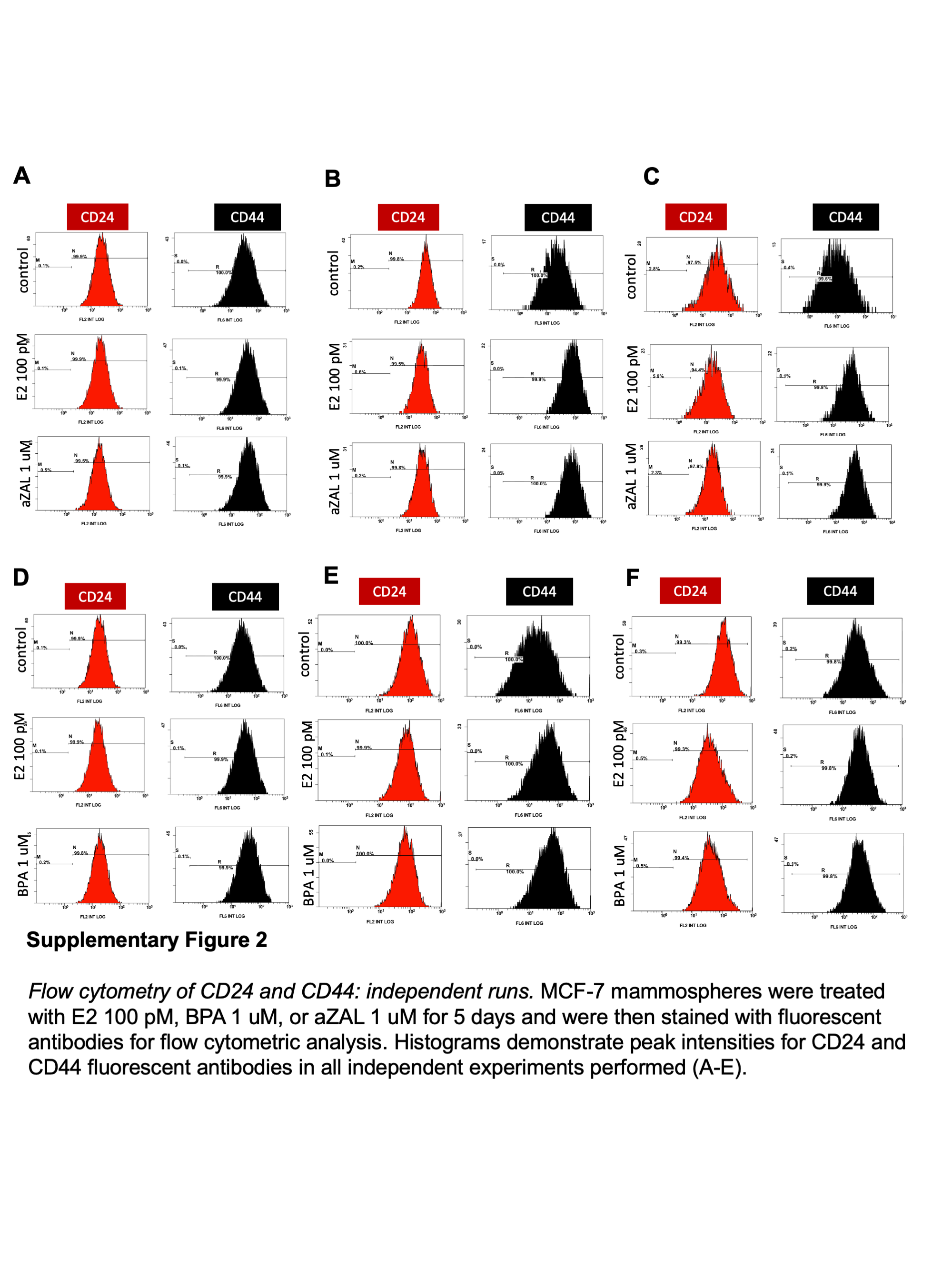

Supplement: Supplementary file 2 — Supporting File 2 [file MC-65-907-s005.tiff]

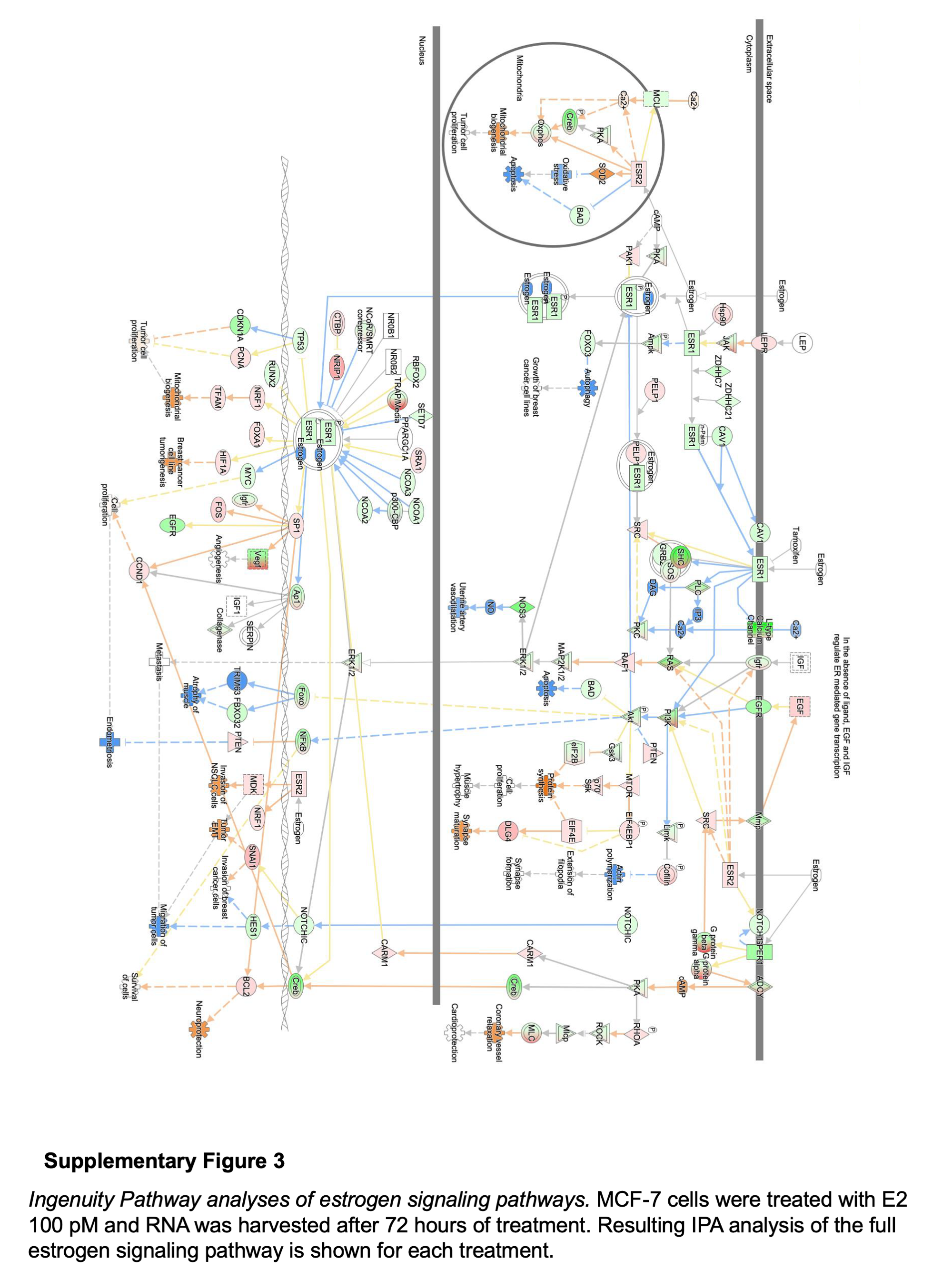

Supplement: Supplementary file 3 — Supporting File 3 [file MC-65-907-s003.tiff]

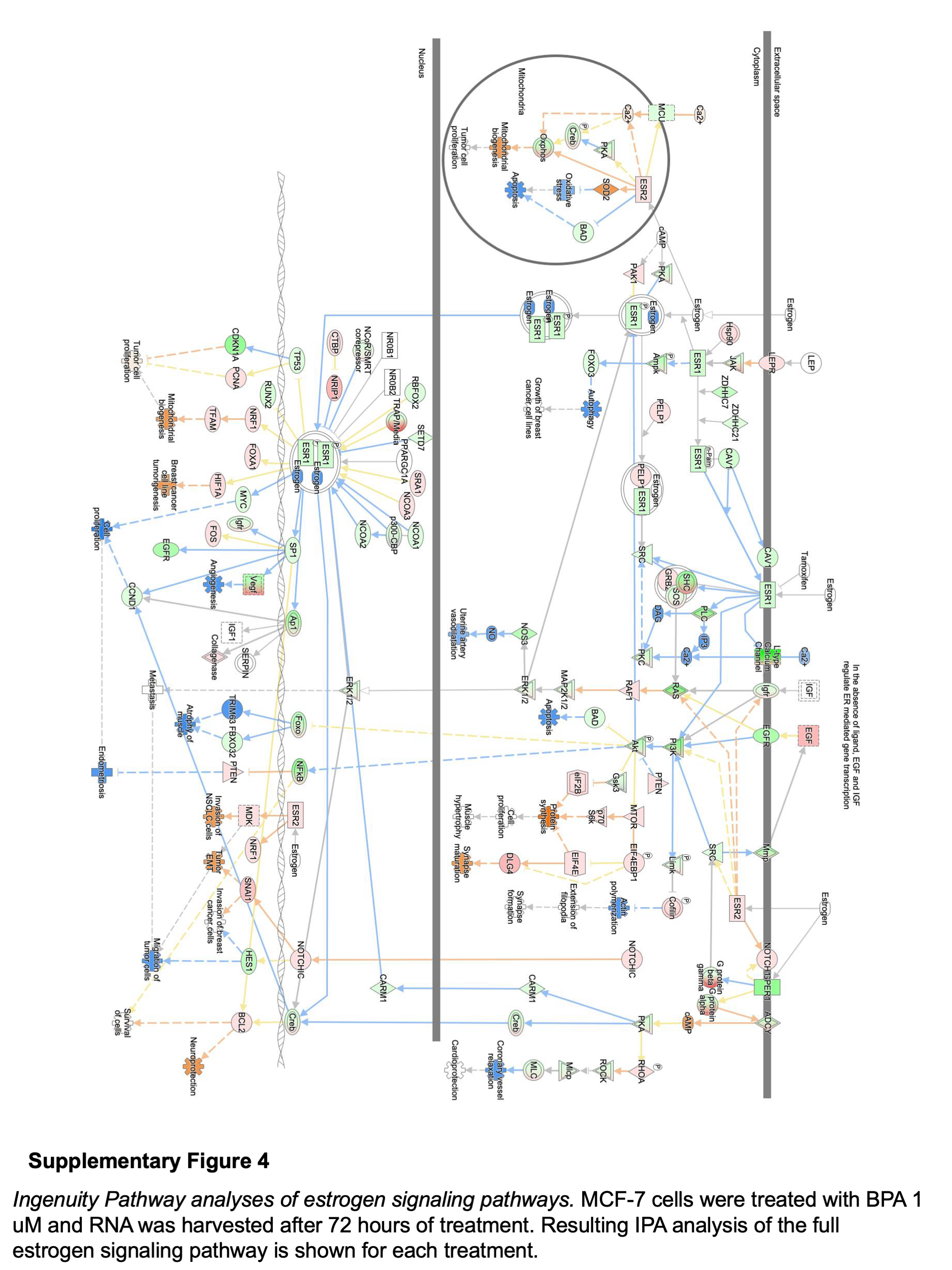

Supplement: Supplementary file 4 — Supporting File 4 [file MC-65-907-s004.tiff]

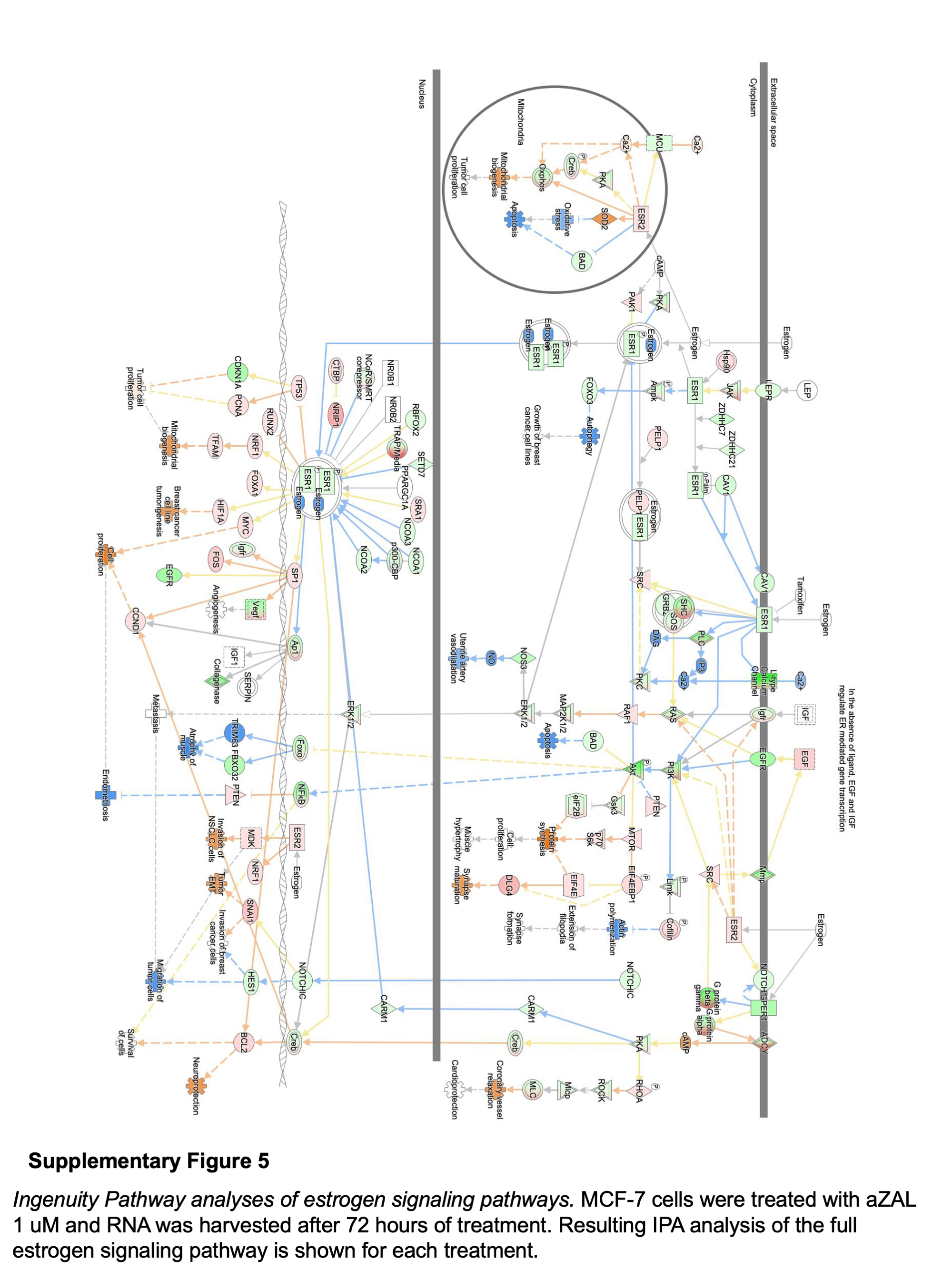

Supplement: Supplementary file 5 — Supporting File 5 [file MC-65-907-s002.tiff]
